# Changes in muscle quality and biomarkers of neuromuscular junctions and muscle protein turnover following 12 weeks of resistance training in older men

**DOI:** 10.5114/biolsport.2024.141064

**Published:** 2024-07-08

**Authors:** Andrzej Mastalerz, Bizhan Hooshmand-Moghadam, Shadi Moazamigoudarzi, Fateme Golestani, Babak Hooshmand-Moghadam, Monika Johne, Krzysztof Maćkała, Katarzyna Lorenz, Ewelina Maculewicz

**Affiliations:** 1 Faculty of Physical Education, Jozef Pilsudski University of Physical Education in Warsaw, Warsaw, Poland; 2 Department of Exercise Physiology, Faculty of Sport Sciences, Ferdowsi University of Mashhad, Mashhad, Iran; 3 Department of Sciences for Quality of Life, University of Bologna, Rimini, Italy; 4 Department of Exercise Physiology, University of Birjand, Birjand, Iran; 5 Department of Track and Field, Wroclaw University of Health and Sport Sciences, Wrocław, Poland

**Keywords:** Resistance Training, Muscle Quality, Neuromuscular Junctions, Protein Turnover, Elderly

## Abstract

The present study aimed to investigate the effects of 12 weeks of resistance training (RT) on body composition [fat mass (FM), lean body mass (LBM)], muscle quality upper and lower (MQU, MQL), muscle size [cross sectional area (CSA), quadriceps cross-sectional area (QCSA)], biomarkers of neuromuscular junctions [C-terminal agrin fragment (CAF)], and muscle protein turnover [N-terminal peptide (P3NP), 3-methylhistidine (3MH), skeletal muscle-specific isoform of troponin T (sTnT)] in older men. Thirty elderly men (age 66.23 ± 0.57 years) were randomly divided into two groups: resistance training group (RT, n=15) and control group (CON, n=15). Participants in RT performed resistance training protocols with the intensity of 60% one-repetition maximum (3 × /week, 4 sets of the six exercise circuits). Blood samples were assessed before (pre-test) and after (post-test) a 12-week intervention. The ANCOVA (2 × 2; group × time; with the baseline variable as the covariate) revealed significant interaction effects; the greater increases for LBM (P < 0.001), CSA (P < 0.001), QCSA (P < 0.001), MQU (P < 0.05), MQL (P < 0.01), 3MH (P < 0.05) were noted in RT than CON, and greater decreases for FM (P < 0.001) and CAF (P < 0.001) in RT than CON. No interaction effect was found for P3NP and sTnT. The time effect was found for all variables besides P3NP in RT, but no time effect was revealed in CON. The 12-week RT was an effective strategy for improving the concentrations of neuromuscular junction biomarkers such as 3-MH and CAF in older adults, and may lead to favourable changes in body composition, muscle quality, and muscle size.

## INTRODUCTION

The aging population is a growing concern worldwide which exposes individuals to the increased risk of a debilitating musculoskeletal condition [1]. The main characteristic of ageing is a notable lack and weakness in muscle mass and strength [2]. Recently, it has gained credence that neurophysiological processes are important in maintaining skeletal muscle health with advancing age [3]. Neuromuscular junction (NMJ) is essential for nerve-to-muscle crosstalk [4]. During the ageing process, NMJ undergoes significant remodelling, whereby the precise alignment between pre-and postsynaptic structures is reduced, leading to a progressive accumulation of denervated muscle fibres [4]. Hence, biomarkers of NMJ stability have been suggested as indicators of skeletal muscle disorder [4].

**Table:** 

Abbreviation	Explanation
**1RM**	One-repetition maximum
**3MH**	3-methylhistidine
**AMPK**	AMP-Activated Protein kinase
**BMI**	Body mass index
**CAF**	C-terminal agrin fragment
**CON**	Control group
**CSA**	Cross sectional area;
**QCSA**	Quadriceps cross sectional area
**FM**	Fat mass
**LBM**	Lean body mass
**MQ**	Muscle quality
**MQU**	Muscle quality upper
**MQL**	Muscle quality lower
**mTOR**	Mammalian target of rapamycin complex 1
**P3NP**	N-terminal peptide
**sTnT**	Skeletal muscle-specific isoform of troponin T
**RT**	Resistance training
**NMJ**	Neuromuscular junction
**PKB**	protein kinase B

C-terminal agrin fragment (CAF) concentrations have been recognized as a key contributing factor of skeletal muscle deterioration [3]. Agrin is a proteoglycan heparin sulfate involved in the development and maintenance of NMJ [4]. It has been reported that higher concentrations of CAF may adversely affect skeletal muscle quality and NMJ. According to previous evidence, older adults with higher concentrations of CAF experienced lower muscle mass and strength [3]. Type III collagen is a subgroup of collagen found in skeletal muscle and synthesized from procollagen type III molecule during cleavage of C and N terminals. During this process, Nterminal propeptide (P3NP) is cleaved and released into the bloodstream [5]. P3NP is recognized as a biomarker for the investigation of skeletal muscle remodelling [5]. Additionally, 3-methylhistidine (3MH) indicates myofibrillar breakdown and is formed by posttranslational methylation of specific histidine residues in the myofibrillar protein actin and myosin [6]. 3-MH concentrations can also be a key factor indicating high levels of Protein turnover [7]. 3-MH concentrations are formed in the muscle through the Post-translational methylation of histidine residues in actin and myosin. During muscle breakdown, 3-MH is released and found in urine [7]. The troponin complex composed of three subunits, is at the centre of Ca^2+^ regulation of muscle contraction including troponin C is the Ca^2+^-binding subunit, the inhibitory subunit is troponin I, and the tropomyosin binding subunit troponin T (TnT) [8, 9]. Since troponins are not normally found in the blood, older adults are more likely to elevate serum concentration of sTnT associated with loss of muscle function and strength [10].

Physical activity promotes muscle remodelling and muscle mass and strength due to mechanical tension on intramuscular connective tissue which increases the activity of satellite cells and changes in collagen synthesis [11]. Resistance training (RT) increases mechanical tension on muscle tissue and its connective tissue [12]. RT can also improve the neuromuscular and functional performance of elderly adults [13]. Pieces of evidence suggested that there is a positive correlation between RT and improvement in NMJ [14]. However, to the best of the authors’ knowledge, limited and contradictory studies have investigated the role of RT on neuromuscular biomarkers and muscle-specific biomarkers [11]. Consequently, considering the effect of RT on active muscle secretions in training and the change of muscle biomarkers due to exercise in the elderly and the availability of these biomarkers, we investigate the effect of resistance training on serum concentrations of CAF, P3NP, 3MH, sTnT in elderly men. Also, we evaluated changes in body composition and muscle size [cross-sectional area (CSA), quadriceps cross-sectional area (Quadriceps CSA)], and muscle quality upper and lower (MQU, MQL) in elderly men.

## MATERIALS AND METHODS

### Participants

**FIG. 1:**
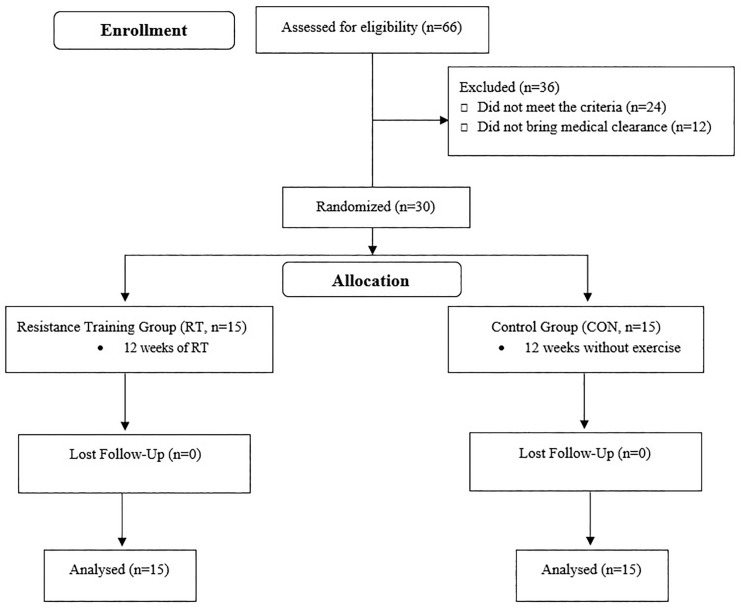
Flowchart of the study.

Thirty elderly men (age 66.23 ± 0.57 years) volunteered to partake in this study. Figure 1 shows the flowchart of this study. Inclusion criteria included: age between 60–80 years, any diseases, no smoking and alcohol, any medication and nutritional supplements, and any history of regular physical activity at least in the past year. Exclusion criteria included having more than three absences during the training sessions, unwillingness to continue cooperation, the presence of diseases, smoking, alcohol consumption, and use of medication or supplementation. Information about the physical activity level and health condition of all the participants was collected via questionnaire. Subjects were fully informed of the procedures and possible risks of the investigation and gave their informed written consent to participate. The study was approved by the ethics committee of the sports science research institution of Iran (code number: IR.SSRC. REC.1398.062, 07/09/2019) and carried out in agreement with the latest version of the Declaration of Helsinki.

### Study design

This was a semi-experimental study with a pre- and post-test design. After completing the informed consent form, the subjects were randomly divided into two groups of resistance training group (RT, N = 15) and the control group (CON, N = 15). The subjects in the RT participated in a 12-week training program based on the recommendation of the American College of Sports Medicine. Participants of CON were asked to maintain their normal daily activities during the study period. Measurements were collected at two time points, baseline and after 12 weeks, during the same time of day (± 1 hr) and under the same environmental conditions (~20°C and ~55% humidity) in the morning following an overnight fast and abstinence from caffeinated drinks, alcohol, and approximately 48 hr after the last training session. Randomization was performed by using a digital tool available at www.randomizer.org. Also, the calculation of the sample size was carried out using G∗Powers software (Heinrich-Heine-Universität, Düsseldorf, Germany).

*Training intervention:* The exercise group participated in the 12-week RT intervention (3 sessions/ week). Table 1 shows the RT program in this study. First, participants of RET warmed-up about 10 minutes of low-intensity treadmill walking. Then, they performed the RT program which contained Leg Press, Chest Press, Seated Row, Leg Extension, Lat Pulldown, and Tricep Dips; and included 15 tempo repetitions of one exercise in 60-sec and 30-sec rest before the start of the next exercise. Participants performed four sets of the six exercise circuits in each session for RT. They started the RT with ~60% of 1RM, but when an exercise was comfortably completed in 60 sec, an ~5% increment was added for the next training session to ensure that a gradual overload was provided during the training intervention. Finally, at the end of RT, participants performed 10 10-minute cool down. All training sessions were conducted between 4 and 6 PM under the supervision of RT specialists. The periodized protocols were adapted from previous studies [13].

**TABLE 1:** Resistance training program (RT)

	Training structure
**Sessions per week**	3
**Warm-up**	10 min treadmill running/ low density
**Resistance training**	**Exercise:** Leg Press, Chest Press, Seated Row, Leg Extension, Lat Pulldown, Tricep Dips **Intensity:** 60% 1RM **Volume:** 1 min per exercise, 30 sec recovery per exercise **Repetition:** Repeat × 4 sets
**Cool-down**	10 min

*Serum parameters:* At two stages, 48 h before the start of the study (pretest) and 48 h after the last training session (post-test), 10 mL blood was obtained by venipuncture (in a heparinized tube) from a cubital vein. In both stages, sampling was carried out after approximately 12 hours of fasting. Blood samples were then centrifuged (at 3000 rpm for 10 min), and serum was extracted and stored at -80°C until the final measurement. Serum levels of CAF (MyBioSource Co, sensitivity: < 28.125 pg/ml), P3NP (Cusabio Co, sensitivity: 0.078 ng/ml), 3MH (MyBioSource Co, sensitivity: < 3.75 nmol/ml), and sTnT (Cusabio Co, sensitivity: 0.039 ng/ ml), were determined using ELISA human kit. Intra and inter-assay CV for all blood markers were less than 8% and 10%, respectively.

*Body Composition measurements:* Participants were instructed to complete an overnight fast of 12 hours, attempt to sleep for at least 8 hours and refrain from physical activity for 48 hours before the test. Upon arriving at the laboratory, participants were asked to void completely within 30 min of the test. Height was measured by using a wall-mounted stadiometer (SECA, Germany). Body mass was evaluated by the digital SECA scale (Germany). Fat mass (FM) and lean body mass (LBM) were measured by a multi-frequency bioelectrical impedance device (Jawon Medical X Contact-356, South Korea. The test-retest reliability of the bioelectrical impedance method is high (R = 0.95 to 0.99) [15].

*Muscle size measurements:* Computed tomography (CT) was used to calculate the cross-sectional area and mass of the knee extensor muscle. Imaging was done by a Siemens 64-slice scanner (Somatom Definition AS; Siemens medical solution, Forchhim, Germany). Subjects laid in the supine position with legs extended. Images were taken at the midpoint between the lateral femoral condyle and the greater trochanter to calculate the lean mass of the knee extensor (middle of the thigh). To calculate lean mass, cross-sectional area images were taken from 1-mm thickness and 0-mm distance from knee to hip. Then, the cross-sectional area and lean mass of the knee extensor were analyzed by Singo software (Singo Leonardo, Siemens, Medical system, Germany) [16]. Lab specialists performed all the measurements.

*Muscle quality:* MQ was determined as the ratio of muscle strength or power and muscle mass as suggested previously [17]. The MQ score of upper extremities was calculated based on handgrip strength measured by hand dynamometer (kg) divided by muscle mass calculated using the BIA equation of Janssen et al. [18]. To calculate power for MQ of lower extremities the equation for peak power (W) [−715.218 + 13.915 × body weight (kg) + 33.425 × stand in 20 s] of Smith et al. [19] predicting lower-body muscle power in older adults using a 30-s chair stand test was used. The calculated power was then divided by muscle mass.

*Nutrition and dietary analysis:* All subjects were encouraged to adhere to their normal and similar dietary patterns throughout the study. Subjects were required to submit 3-day (2 weekdays and 1 weekend) food records three times (Before and at the end of week 6 and week 12). Each food item was individually entered into the nutritionist software v.4 and total energy consumption, as well as the amount of energy derived from proteins, fats, and carbohydrates, were analyzed [13].

*Statistical analysis:* The normality of data distribution was evaluated by the Shapiro–Wilk test. Analysis of covariance (ANCOVA with the baseline variable as the covariate) was applied to examine the intra- and inter-group differences, respectively. A paired-sample t-test was applied to examine post-hoc effects. Statistical significance was set at P ≤ 0.05. Statistical significance was set at P ≤ 0.05. All statistical procedures were analyzed using the statistical package for social sciences (SPSS Inc., Chicago, IL, USA) software version 22 and were expressed as means ± SD and all the figures were prepared in Graphpad Prism (version 8.0.2).

## RESULTS

*Dietary Intake Monitoring and Compliance with Exercise Training:* There were no reports of an adverse event from our training interventions. The mean demographic characteristics of the two groups are summarized in Table 2. Dietary analysis showed, there were no significant differences between energy intake, proteins, fats, and carbohydrates consumed between RT and CON (Table 3). Overall adherence to exercise training was 100%.

**TABLE 2:** Demographic data of participants

Variable	CON group (n=15)	RT group (n=15)
Age (year)	66.13 ± 3.04	66.33 ± 3.35
Height (cm)	168.25 ± 3.33	167.93 ± 3.59
Weight (kg)	61.85 ± 3.79	64.85 ± 3.74

Abbreviation: Values are presented as Mean ± SD; CON: control; RT: resistance training.

**TABLE 3:** Energy and macronutrients at the before and at the end of week 6 and week 12. Values are Mean ± SD.

	Group	Baseline	6 weeks	12 weeks	Group differences (P)
Energy (kcal/day)	CON	1800.00 ± 75.26	1774.53 ± 53.92	1771.46 ± 46.66	0.58
	RT	1773.33 ± 49.77	1799.46 ± 54.10	1792.80 ± 56.85	

Protein (g/day)	CON	77.66 ± 4.28	79.06 ± 5.32	80.20 ± 5.26	0.30
	RT	80.40 ± 7.47	82.53 ± 6.86	77.80 ± 4.72	

Carbohydrate (g/day)	CON	244.53 ± 9.19	247.26 ± 7.86	246.26 ± 6.72	0.68
	RT	242.93 ± 6.95	247.33 ± 7.94	249.80 ± 6.85	

Fat (g/day)	CON	56.80 ± 8.04	52.13 ± 3.48	51.73 ± 3.08	0.89
	RT	53.33 ± 2.76	53.33 ± 3.17	53.60 ± 3.71	

Abbreviation: CON – control; RT – resistance training. P– statistical significance of differences between resistance training (RT) and control (CON) groups.

**FIG. 2:**
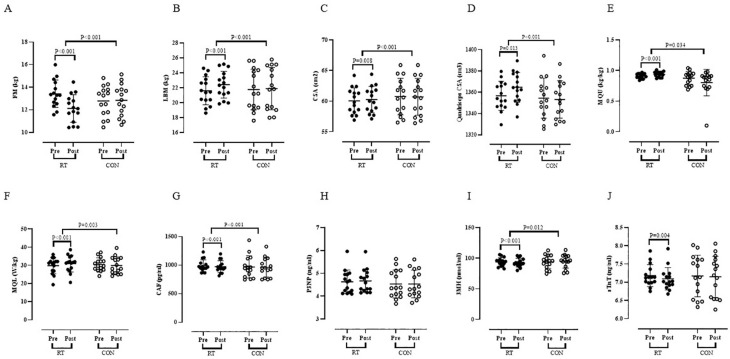
Body composition, muscle size, muscle quality and blood markers before (Pre) and after 12 weeks of intervention (Post) in control (CON; white circles) and resistance training groups (RT; black circles). Abbreviation: A – fat mass, FM; B – lean body mass, LBM; C – cross sectional area, CSA; D – quadriceps cross sectional area; E – muscle quality upper, MQU; F – muscle quality lower, MQL; G – C-terminal agrin fragment, CAF; H – N-terminal peptide, P3NP; I – 3-methylhistidine, 3MH and J – skeletal muscle-specific isoform of troponin T, sTnT.

*Body Composition, Muscle size and Muscle quality:* Results indicated a significant decrease for FM (P < 0.001) (Figure A) in RT group vs. pretest. In addition, there were significant increases for LBM (P < 0.001) (Figure B), CSA (P = 0.008) (Figure C), quadriceps CSA (P = 0.013) (Figure D), MQU (P < 0.001) (Figure E), and MQL (P < 0.001) (Figure F) in RT group vs. pretest. Based on the results of the present study, there were significant alterations in RT group vs. CON group for FM (P < 0.001), LBM (P < 0.001), CSA (P < 0.001), quadriceps CSA (P < 0.001), MQU (P = 0.034), and MQL (P = 0.003) (Figure 2 A, B, C, D, E, and F, respectively).

*Blood Markers:* Results of the present study found a significant reduction in serum concentrations of CAF (P < 0.001) (Figure G), 3MH (P < 0.001) (Figure I), and sTnT (P = 0.004) (Figure J) in the RT group vs. pretest. RT group showed significant differences in serum concentrations of CAF (P < 0.001) and 3MH (P = 0.012) vs. CON group (Figure G and I, respectively). There were insignificant changes between RT group vs. CON group in serum concentrations of sTnT (P = 0.079) (Figure J). However, serum concentrations of P3NP observed neither a within-group nor a between-group significant alterations after 12 weeks of intervention (*P* > 0.05) (Figure H).

## DISCUSSION

In this semi-experimental study, we evaluated the effects of 12 weeks of RT on biomarkers of neuromuscular junctions, muscle size, muscle quality upper and lower, and body composition in older adult men. The main findings of the present study indicated that 12 weeks of RT induced meaningful alterations in serum concentrations of CAF and 3MH in older adult men. Furthermore, results showed insignificant inter-group and intra-group differences in serum concentrations of P3NP in older adult men. In addition, the results indicated a significant effect of RT on improving body FM, LBM, CSA, quadriceps CSA, MQU, and MQL in older adult men.

Based on our results, we found a significant reduction in serum concentrations of CAF after 12 weeks of RT in elderly men. By ageing, NMJ decline occurs in both animal and human models [20]. Recently, higher circulating concentrations of CAF have emerged as a potential biomarker of skeletal muscle deterioration, due to its inverse relationship with NMJ health [4]. There are limited studies carried out on the effect of RT on CAF concentrations. In the present study, the observed significant reduction in CAF was in agreement with the study of Bigdeli et al. [11] which showed that six weeks of functional training with blood flow restriction induced a significant reduction in serum concentrations of CAF in older adult men. Drey et al. [21] showed a significant reduction in serum CAF concentrations following 20 weeks of power and strength training in older adults. However, Bigdeli et al. [11] reported that a 6-week functional and blood flow restriction training could not decrease the serum concentrations of CAF in healthy older men. Our results are contradictory to the findings of Fragala et al. [12] who reported that a 6-week RT with 70% 1-RM induced a significant increase in serum concentrations of CAF in older adult men and women. Perhaps, intensity, duration, and type of exercise could explain the contradictory agreements in literature to our findings. Although the mechanisms responsible for the reduction of CAF following 12 weeks of RT remain unknown. Also, it is well demonstrated that neuromuscular adaptation which occurs in the early stage of RT results in a reduction of serum concentration CAF [11]. Neural adaptation is explained by an increase in agonist activation, motor unit recruitment, and firing frequency against NMJ fragmentation [22]. Based on the results of Deschenes et al. [23] who suggested NMJ fragmentation may decrease by neuromuscular activity and against agerelated NMJ degradation. In the present study, along with a significant reduction in serum concentrations of CAF, LBM significantly increased in RT group. Based on the previous evidence, there is a tightly inverse correlation between the circulating of CAF and LBM [24]. Therefore, based on our results, one possible explanation is that the reduction in serum concentrations of CAF was dependent on subsequent changes in LBM. Overall, RT appears to be an effective and well-tolerated strategy for improving markers of CAF in older adult men.

In terms of effect of RT on P3NP, our findings showed an insignificant increase in serum concentrations of P3PN following of 12 weeks of RT group vs. CON in adult men. There are limited studies carried out on the effect of RT on P3NP. In agreement with our finding, Fragala et al. [5] showed an insignificant increase in serum concentrations of P3NP following six weeks of RT. However, our findings were contradictory to the results of Bhasin et al. [25] who reported a significant enhancement in serum concentrations of P3NP following four weeks of anabolic treatment of testosterone and recombinant growth hormones in older men. Pieces of evidence indicated that concentrations of P3NP are responsive to exercise, testosterone, and growth hormone (GH) [25]. Also, an increase in P3NP concentrations depends on enhancement in concentrations of GH and testosterone following exercise training [26]. However, serum concentrations of testosterone and GH have not been evaluated in the present study, perhaps due to the age of participants who had lower levels of testosterone. During Type III collagen synthesis, the P3NP is cleaved and released into circulation in direct proportion to type III collagen synthesis [5]. The cleaved P3NP results from the proteolytic cleavage in the synthesis of skeletal muscle that can be measured in the blood [5]. Since it is a product of synthetic pathways, P3NP is reflective of actual tissue remodelling [5]. On the other hand, increases in concentrations of P3NP are depended on type III collagen peptide which contributes to the growth of myoblasts and muscle formation and is caused by muscle hypertrophy [27]. Perhaps the intensity or duration recruited in the present study was not sufficient for the synthesis of structural proteins.

Based on previous studies, circulations of 3-MH are associated with the reduction of LBM and enhancement of FM in older adults [7]. The present study demonstrated a significantly greater decrease in serum concentrations of 3-MH in the RT group compared to the CON group. Results from previous studies have been inconsistent in the study of Yarasheski et al. [28] in which reported that no significant alterations in urinary concentrations of 3-MH after 12-week RT in healthy older individuals. Frontera et al. [29] showed that 12 weeks of RT increased urinary concentrations of 3-MH in older adults. Maybe the method of exercise, intensity and duration of intervention, and the type of participants are contradictory reasons to our results. Additionally, a reduction in serum concentrations of 3-MH may have been associated with an increase in LBM, MQ, and CSA which supported previous pieces of evidence in which showed high concentrations of 3-MH associated with notable lack and weakness in muscle mass [7]. Based on the results of the previous research, it has been indicated that RT lead to an increase in muscle protein fractional synthesis and breakdown which increases in muscle fibre type of I and II and ultimately muscle hypertrophy [29]. Therefore, in the present study, RT may lead to the maintenance of positive net protein balance which is against the age-related loss of skeletal muscle protein.

sTnT is one of the proteins of contractile machinery which may be used as a marker for sarcomere dysfunction and muscle wasting [8]. Serum levels of sTnT can leak out to the blood following the aging process as well as a skeletal muscle injury [10]. Concentrations of sTnT can used as an index for evaluating the effect of physical exercise on muscle function [30]. King et al. [31] illustrated that there was an association between the increase in strength and a drop in serum concentrations of sTnT. Based on to the best of authors’ knowledge, the present study was the first one that investigated the effect of 12 weeks of RT in serum concentration of sTnT in elderly adult men. In this respect, Banitalebi et al. [32] reported no significant changes in serum concentrations of sTnT in women with osteosarcopenic obesity after performing 12 weeks of elastic band resistance training. Huxley and Simmons reported that 10-week strength training elicited a substantial decrease in serum concentrations of sTnT, paralleled by improvements in physical performance and muscle strength in older community-dwellers [33]. In the present study, despite significant enhancement in MQ and LBM, no significant changes were observed in serum concentrations of sTnT. In connection with the different results in our study versus previous studies, there was a possibility that the response of sTnT may be different depending on the intensity or type of exercise training. The participants in this study performed RT program with an intensity of 60% 1RM which was not sufficient for changes in the serum concentration of sTnT.

Data from the present study also showed that participants in RT group experienced significant improvement in LBM and MQ (both upper and lower) after 12 weeks of intervention. Similarly, Brooks et al. [34] reported that 16 weeks of strength training significantly improved MQ and type II fibre cross-sectional area in older adults with type 2 diabetes. In another study by Vikberg et al. [35] it was demonstrated that 10 weeks of functional training in older men led to an increase in LBM and muscle function. Additionally, Murlasits et al. [36] reported eight weeks of RT significantly increased LBM and MQ in women and men. Exercise training has been widely indicated as a useful treatment to counter or attenuate ageing-related impairments in LBM and MQ [37]. The main mechanisms through which RT may improve LBM and MQ are complex. However, qualitative improvements in muscle appear to account for neuromuscular adaptation more than muscle hypertrophy. More specifically, RT increases the major contractile protein synthesis such as myosin heavy chain (MHC) and actin, and also shifts the expression of MHC isoforms from MHC IIb to MHC IIa [38].

The findings of the present study demonstrated significant improvement in CSA and quadriceps CSA in RT group vs. CON group. These results are in agreement with the findings of Luciano et al. [39] who showed that quadriceps CSA were significantly increased following 12 weeks of three models of RT (endurance RT, strength RT, and hypertrophy RT) in Wistar rats. Also, Nilwik et al. [40] demonstrated that 6 months of resistance training-induced significant improvement in quadriceps muscle CSA both in young and elderly men. RT results in high activation of protein kinase B (PKB), which is responsible for the phosphorylation of TSC2 at the Thr1462 site, resulting in TSC2 inactivation and consequently promoting mammalian target of rapamycin complex 1 (mTOR) activation [39]. It is known that this pathway is necessary for inducing muscle hypertrophy once the inhibition by rapamycin induces muscle atrophy [39]. It has been demonstrated that energy production during RT increases the activation of adenosine monophosphate-activated protein kinase and eukaryotic translation initiation factor-4E-binding protein 1 (4E-BP1) which (mTORC1) signalling; a potential kinase stimulates muscle hypertrophy [39]. So, the increase in CSA and quadriceps CSA after 12 weeks of RT intervention might be due to the mTORC1 signalling cascade.

We acknowledged that the current study had methodological limitations. Only a single 48-h collection was analyzed under each condition; due to the different time course of the change in muscle-specific biomarkers and non-muscle-specific biomarkers, more research is needed. Moreover, since ethical considerations in human samples (older adults), the variables were measured through blood samples and it was not possible to measure the variables by biopsy. Therefore, further studies are recommended to generalize the results due to the limitations of this study.

## CONCLUSIONS

According to the results of the present study, RT was an effective strategy for improving concentrations of neuromuscular junction biomarkers such as 3-MH and CAF in older adults and may lead to favorable changes in body composition, muscle quality, and muscle size. Our results provide early insights into responses of circulating neuromuscular junction biomarkers to intervention and also support further research efforts to better elucidate potential responses and clinical.

## Data Availability

The data that support the findings of this study are available from the corresponding author upon reasonable request. Some data may not be made available because of privacy or ethical restrictions.
